# Treating PTSD: A Review of Evidence-Based Psychotherapy Interventions

**DOI:** 10.3389/fnbeh.2018.00258

**Published:** 2018-11-02

**Authors:** Laura E. Watkins, Kelsey R. Sprang, Barbara O. Rothbaum

**Affiliations:** Department of Psychiatry and Behavioral Sciences, Emory University School of Medicine, Atlanta, GA, United States

**Keywords:** posttraumatic stress disorder, psychotherapy, treatment, evidence-based medicine, prolonged exposure, cognitive processing therapy, cognitive behavioral therapy

## Abstract

Posttraumatic stress disorder (PTSD) is a chronic, often debilitating mental health disorder that may develop after a traumatic life event. Fortunately, effective psychological treatments for PTSD exist. In 2017, the Veterans Health Administration and Department of Defense (VA/DoD) and the American Psychological Association (APA) each published treatment guidelines for PTSD, which are a set of recommendations for providers who treat individuals with PTSD. The purpose of the current review article is to briefly review the methodology used in each set of 2017 guidelines and then discuss the psychological treatments of PTSD for adults that were strongly recommended by both sets of guidelines. Both guidelines strongly recommended use of Prolonged Exposure (PE), Cognitive Processing Therapy (CPT) and trauma-focused Cognitive Behavioral Therapy (CBT). Each of these treatments has a large evidence base and is trauma-focused, which means they directly address memories of the traumatic event or thoughts and feelings related to the traumatic event. Finally, we will discuss implications and future directions.

## Introduction

Posttraumatic stress disorder (PTSD) is a chronic, often debilitating mental health disorder that may develop after a traumatic life event, such as military combat, natural disaster, sexual assault, or unexpected loss of a loved one. Most of the U.S. population is exposed to a traumatic event during their lifetime (Sledjeski et al., 2008) and shortly after exposure, many people experience some symptoms of PTSD. Although among most individuals these symptoms resolve within several weeks, approximately 10%–20% of individuals exposed to trauma experience PTSD symptoms that persist and are associated with impairment (Norris and Sloane, 2007). Lifetime and past year prevalence rates of PTSD in community samples are 8.3% and 4.7%, respectively (Kilpatrick et al., 2013), with similar rates (8.0% and 4.8%) observed in military populations (Wisco et al., 2014). PTSD is associated with a wide range of problems including difficulties at work, social dysfunction and physical health problems (Alonso et al., 2004; Galovski and Lyons, 2004; Smith et al., 2005). Fortunately, effective psychological treatments for PTSD exist.

### Diagnostic Criteria

The diagnosis of PTSD has undergone a number of changes since it was initially included in the Diagnostic and Statistical Manual of Mental Disorders Third Edition (DSM-III; American Psychiatric Association, 1980), including a revision in the most recent edition released in 2013 (DSM-5; American Psychiatric Association, 2013). Because the majority of PTSD treatment research currently published used criteria from the DSM-Fourth Edition-Text Revision (DSM-IV-TR; American Psychiatric Association, 2000) or from an earlier version of the DSM, it is important to note how the DSM-5 differs from these earlier versions. The DSM-5 reclassified PTSD as a Trauma- and Stressor-Related Disorder instead of an Anxiety Disorder. In the initial formulation of PTSD, a traumatic stressor was defined as an event outside the range of usual human experience. However, with recognition that traumatic events are relatively frequent, this criterion was revised. DSM-IV and DSM-IV-TR required that intense fear, helplessness, or horror were present in the individual’s response to the traumatic event, although it became evident that this was not universal, especially in military populations. The DSM-5 increased specification as to what qualifies as a traumatic event (Criterion A) and conceptualized traumatic events as exposure to actual or threatened death, serious injury, or sexual violation, as directly experiencing traumatic events, learning of the traumatic events experienced by a close family member or close friend, or repeated exposure to aversive details of the traumatic events. DSM-5 removed the requirement that intense fear, helplessness, or horror were present in the individual’s response to the traumatic event.

The symptom clusters of PTSD also have been revised in DSM-5. DSM-III and DSM-IV included three symptom clusters (re-experiencing, avoidance/numbing and arousal). DSM-5 transitioned from the original three symptom clusters to four symptom clusters including intrusion (five symptoms, one or more required for diagnosis), avoidance (two symptoms, one or more required for diagnosis), negative alteration in cognition and mood associated with the traumatic event (seven symptoms, two or more required for diagnosis) and marked alterations in arousal and reactivity associated with traumatic events (six symptoms, two or more required for diagnosis). The increase to four symptom clusters was a result of splitting avoidance/numbing into distinct clusters (avoidance and negative alteration in mood and cognition). In addition, negative alteration in mood and cognition contains symptoms previously considered numbing symptoms as well as persistent negative emotional states. Marked alterations in arousal and reactivity maintains symptoms previously considered arousal symptoms, in addition to irritable or aggressive behavior and reckless or self-destructive behavior. Consistent with previous editions of the DSM, these symptoms must be present for more than 1 month, cause clinically significant distress or impairment, and not be attributable to substance use or another medical condition. Familiarity with the DSM symptoms of  PTSD is important for two primary reasons: diagnosing PTSD and understanding what traumatic event will be the focus of therapy. “Rape victim” or “combat veteran” is not a diagnosis. Before commencing psychological treatment for PTSD, the provider must be assured that PTSD is primary. When the patient presents with multiple traumatic events, current re-experiencing symptoms will often point towards what we refer to as the “index trauma,” which will be the focus of psychological therapy.

### PTSD Treatment Guidelines

A number of psychological treatments for PTSD exist, including trauma-focused interventions and non-trauma-focused interventions. Trauma-focused treatments directly address memories of the traumatic event or thoughts and feeling related to the traumatic event. For example, both Prolonged Exposure (PE) and Cognitive Processing Therapy (CPT) are trauma-focused treatments. Non-trauma-focused treatments aim to reduce PTSD symptoms, but not by directly targeting thoughts, memories and feelings related to the traumatic event. Examples of non-trauma-focused treatments include relaxation, stress inoculation training (SIT) and interpersonal therapy. Over the last two decades, numerous organizations (e.g., American Psychiatric Association, 2004; National Institute for Health and Clinical Excellence, 2005; Institute of Medicine, 2007; ISTSS [Foa et al., 2009]) have produced guidelines for treatment of PTSD, including guidelines by American Psychological Association (APA) and the Veterans Health Administration and Department of Defense (VA/DoD) that were both published in 2017. Guidelines are lengthy and contain a great amount of information. Thus, the purpose of the current review is to briefly review the methodology used in each set of 2017 guidelines and then discuss the psychotherapeutic treatments of PTSD for adults that were strongly recommended by both sets of guidelines. The guidelines recommended several medications for treatment of PTSD, such as Sertraline, Paroxetine, Fluoxetine, Venlafaxine (see American Psychological Association, 2017; VA/DoD Clinical Practice Guideline Working Group, 2017) however, for the purposes of this review we will focus solely on psychotherapy. The combination of psychotherapy and medication is not recommended by either these guidelines.

In 2017, the VA/DoD and APA each published a treatment guideline for PTSD. Guidelines for PTSD treatment are a set of recommendations for providers who treat individuals with PTSD. Guidelines are not standards, which are requirements or mandatory. Each of these guidelines was based on systematic reviews of the literature examining treatments for PTSD to recommend treatments with the largest and strongest evidence base. The APA guideline is specifically for treatment of PTSD among adults, while the VA/DoD guideline focuses on recommendations for general clinical management, diagnosis and assessment and treatment for providers working within the VA or DoD.

The APA guidelines (American Psychological Association, 2017) are based on a systematic review conducted by the Research Triangle Institute—University of North Carolina Evidence-Based Practice Center (RTI-UNC EPC; Jonas et al., 2013) and fully follow the Institute of Medicine (IOM; now the National Academy of Medicine) standards for developing high quality, independent and reliable practice guidelines (Institute of Medicine, 2011a,b). The review conducted by RTI-UNC included trials published prior to May 2012. The APA panel consisted of individuals from a number of backgrounds, including consumers, psychologists, social workers, psychiatrists and general medicine practitioners. The APA panel considered four factors in their recommendations: (1) overall strength of the evidence for the treatment; (2) the balance of benefits vs. harms or burdens; (3) patient values and preferences for treatment; and (4) the applicability of evidence to various populations.

The VA/DoD guideline (VA/DoD Clinical Practice Guideline Working Group, 2017) is an update to the 2010 PTSD clinical practice guidelines published by the VA/DoD. This update follows the Guideline for Guidelines, which is an internal document of the VA/DoD Evidence-Based Practice Working Group (2013). Work group members had specialties and clinical areas of interest in ambulatory care, behavioral health, clinical pharmacy, clinical neuropsychology, family medicine, nursing, pharmacology, pharmacy, psychiatry and psychology. A focus group of patients was held prior to finalizing the key questions for the evidence review. The Lewin Team, including The Lewin Group, Duty First Consulting, ECRI Institute and Sigma Health Consulting, LLC, was contracted by the VA and DoD to support development of the guidelines and to conduct an evidence review. The literature review focused on interventional studies published between March 2009 and March 2016. The VA/DoD guideline used the Grading of Recommendations Assessment, Development and Evaluation (GRADE) system to assess the quality of the evidence base and assign a grade for the strength of each recommendation. This system uses four domains to assess strength of each recommendation: (1) balance of desirable and undesirable outcomes; (2) confidence in the quality of the evidence; (3) patient or provider values and preferences; and (4) other implications as appropriate (e.g., resource use, equity, acceptability, feasibility, subgroup considerations).

The recommendations of these two sets of guidelines were mostly consistent. See Table 1 for an overview of the “strongly recommended” and “recommended” treatments for adults with PTSD. Both guidelines strongly recommended use of PE, CPT and trauma-focused Cognitive Behavioral Therapy (CBT). The APA strongly recommended cognitive therapy (CT). The VA/DoD recommended eye movement desensitization therapy (EMDR; APA “suggests”), brief eclectic psychotherapy (BET; APA suggests), narrative exposure therapy (NET; APA suggests) and written narrative exposure. In our discussion of PTSD treatments, we will focus on treatments that were strongly recommended by both guidelines, which includes PE, CPT and CBT. First, we will describe each treatment and evidence for its use and then we will discuss dropout, side effects and adverse effects of these treatments together.

**Table 1:** Clinical practice guidelines for treatment of posttraumatic stress disorder (PTSD).

Clinical practice guideline	Methodology	Strongly recommended therapies	Recommended therapies
American Psychological Association (2017)	Independent systematic review; RCTs published from 5/25/12-6/1/16; Expert Review	CBT, CPT, PE, CT	BEP, EMDR, NET
VA/DoD Clinical Practice Guideline Working Group (2017) (revision of 2010 guidelines)	Independent systematic Review; RCTs published 1/1/09-March 2016; Expert Review	PE, CPT, EMDR, specific CBT for PTSD, BEP, NET and written narrative exposure	SIT, PCT, IPT

*Note. CBT, Cognitive Behavioral Therapy; CPT, Cognitive Processing Therapy; PE, Prolonged Exposure; CT, Cognitive Therapy; EMDR, Eye Movement Desensitization Therapy; BET, Brief Eclectic Psychotherapy; NET, Narrative Exposure Therapy; SIT, Stress Inoculation Training; PCT, Present-Centered Therapy; IPT, Interpersonal Psychotherapy*.

## Strongly Recommended Treatments

### Prolonged Exposure

PE is strongly recommended by both the APA and VA/DoD guidelines for treatment of PTSD. PE is based on emotional processing theory (Foa and Kozak, 1985, 1986), which suggests that traumatic events are not processed emotionally at the time of the event. Emotional processing theory suggests that fear is represented in memory as a cognitive structure that includes representations of the feared stimuli, the fear responses, and the meaning associated with the stimuli and responses to the stimuli. Fear structures can represent realistic threats, which is normal. However, fear structures can become dysfunctional. According to Foa and Kozak (1986), fear structures may become problematic when the association between stimulus elements do not accurately reflect the real world, physiological and escape or avoidance responses are induced by innocuous stimuli, responses that are excessive and easily triggered interfere with adaptive behavior, and safe stimulus and response elements are incorrectly associated with threat or danger. PE focuses on altering fear structures so that they are no longer problematic. Two conditions are necessary for fear structures to be altered and for exposure to work. First, the fear structure must be activated and second, new information that is incompatible with erroneous information in the fear structure must be incorporated into the structure.

The evidence-based manual describing PE indicates that this therapy is typically completed in 8–15 sessions (Foa et al., 2007). PE includes psychoeducation about PTSD and common reactions to trauma, breathing retraining, and two types of exposure: *in vivo* exposure and imaginal exposure. During psychoeducation, patients learn about PTSD, common reactions to trauma and exposure. Breathing retraining is a skill taught to assist patients in stressful situations but not to be used during exposure. The two main components of treatment are *in vivo* exposure and imaginal exposure. *In vivo* exposure assists patients in approaching situations, places and people they have been avoiding because of a fear response due to the traumatic event repeatedly until distress decreases. Imaginal exposure consists of patients approaching memories, thoughts and emotions surrounding the traumatic event they have been avoiding. Patients recount the narrative of the traumatic event in the present tense repeatedly and tape record this recounting to practice imaginal exposure for homework. The patient and therapist then process emotional content that emerged during the imaginal exposure. Through these two types of exposures, patients activate their fear structure and incorporate new information. PE is a particular program of exposure therapy that has been adopted for dissemination through the VA and DOD. The treatment manual has been translated into about nine different languages. A revised PE manual is due to be published in 2019. It has been shown to be helpful across survivors, in different cultures and countries, regardless of the length of time since traumatization or the number of previous traumatic events (Powers et al., 2010).

As suggested by its strong recommendation by both set of guidelines, there is a large body of research evidence that indicates the effectiveness of exposure therapy and particularly PE. Individuals randomly assigned to exposure therapy have significantly greater pre- to posttreatment reductions in PTSD symptoms compared to supportive counseling (Bryant et al., 2003; Schnurr et al., 2007), relaxation training (Marks et al., 1998; Taylor et al., 2003) and treatment as usual including pharmacotherapy (Asukai et al., 2010). In addition to the RCTs used to determine recommended treatment in the guidelines, several meta-analyses have found that exposure therapy is more effective that non-trauma focused therapies (Bradley et al., 2005; Powers et al., 2010; Watts et al., 2013; Cusack et al., 2016). A meta-analysis on the effectiveness of PTSD found the average PE-treated patient fared better than 86% of patients in control conditions on PTSD symptoms at the end of treatment (Powers et al., 2010). The effect sizes for PE were not moderated by time since trauma, publication year, dose, study quality, or type of trauma. A second meta-analysis, which examined psychological treatments for PTSD, found a high strength of evidence for the efficacy of PE (Cusack et al., 2016). Regarding loss of diagnosis, rates vary across studies. Among PE participants, 41% to 95% lost their PTSD diagnosis at the end of treatment (Jonas et al., 2013). In addition, 66% more participants treated with exposure therapy achieved loss of PTSD diagnosis than in waitlist control groups (Jonas et al., 2013).

### Cognitive Processing Therapy

In addition to PE, CPT is strongly recommended by both the APA and VA/DoD guidelines for treatment of PTSD. CPT is a trauma focused therapy drawing on social cognitive theory and informed emotional processing theory as discussed above Resick and Schnicke (1992). CPT assumes that following a traumatic event, survivors attempt to make sense of what happened, often time leading to distorted cognitions regarding themselves, the world, and others. In an attempt to integrate the traumatic event with prior schemas, people often assimilate, accommodate, or over-accommodate. Assimilation is when incoming information is altered in order to confirm prior beliefs, which may result in self-blame for a traumatic event. An example of assimilation is “because I didn’t fight harder, it is my fault I was assaulted.” Accommodation is a result of altering beliefs enough in order to accommodate new learning (e.g., “I couldn’t have prevented them from assaulting someone”). Over-accommodation is changing ones beliefs to prevent trauma from occurring in the future, which may result in beliefs about the world being dangerous or people being untrustworthy (e.g., “because this happened, I cannot trust anyone”). CPT allows for cognitive activation of the memory, while identifying maladaptive cognitions (assimilated and over-accommodated beliefs) that have derived from the traumatic event. The main aim of CPT is to shift beliefs towards accommodation (Resick and Schnicke, 1992).

Resick et al. (2017) have developed an updated treatment manual for CPT. CPT consists of 12 weekly sessions that can be delivered in either individual or group formats. Generally, CPT is composed of CT and exposure components (Resick and Schnicke, 1992; Chard et al., 2012). Clients work to identify assimilated and over-accommodated beliefs and learn skills to challenge these cognitions through daily practice (Resick et al., 2002). Initial sessions are focused on psychoeducation about the cognitive model and exploration of the patient’s conceptualization of the traumatic event. The individual considers: (1) why the traumatic event occurred; and (2) how it has changed their beliefs about themselves, the world and others regarding safety, intimacy, trust, power/control and esteem. The original version of CPT included a written trauma account where the patient described thoughts, feelings and sensory information experienced during the traumatic event. However, following evidence from recent dismantling studies, the most recent version of the protocol does not include the written trauma narrative (Resick et al., 2008, 2017; Chard et al., 2012). CT skills are introduced through establishing the connection between thoughts, feelings, and emotions related to the individual’s stuck points (maladaptive cognitions about the event) and learning ways to challenge cognitions that are ineffective (Chard et al., 2012). These skills are used to examine and challenge their maladaptive beliefs. CPT concludes with an exploration on the shifts in how the individual conceptualizes why the traumatic event occurred, focusing on the shift to accommodation rather than assimilation and over-accommodation.

CPT has been widely supported as an effective treatment for PTSD. While CPT was developed to treat survivors of rape (Resick and Schnicke, 1992), it has been researched and implemented successfully across trauma types and populations (Chard et al., 2012). Research findings suggest CPT effectively treats PTSD in sexual assault survivors (Chard, 2005), veterans who served in Vietnam, Iraq and Afghanistan (Chard et al., 2010), and adult males with comorbid TBI and PTSD (Chard et al., 2011). CPT has been found to exhibit clinically meaningful reduction in PTSD, depression and anxiety in sexual assault and Veteran samples, with results maintained at 5 and 10 year post treatment follow-up (Resick et al., 2012). Meta-analyses suggest that CPT is effective in significantly reducing PTSD symptoms (Watts et al., 2013; Cusack et al., 2016). Similar to findings for PE, the number of individuals who no longer meet criteria for PTSD after CPT varies across studies. Rates of participants who no longer met PTSD diagnosis criteria ranged from 30% to 97% and 51% more participants treated with CPT achieved loss of PTSD diagnosis, compared to waitlist, self-help booklet and usual care control groups (Jonas et al., 2013).

### Cognitive Behavioral Therapy for PTSD

Another strongly recommended therapy by APA and the VA/DoD is CBT for PTSD. The VA/DoD includes only trauma-focused CBT. APA included both trauma-focused and non-trauma-focused CBT in its recommendations including CBT-mixed, which included studies using cognitive behavioral techniques that did not fit in well with other categories, and CT, which included CT studies that were not specifically CPT. Brief trauma-focused CBT categorized by the VA/DoD included studies examining trauma-focused cognitive and/or behavioral techniques that were not specifically PE or CPT. Thus in this section, we will discuss brief therapies using trauma-focused behavioral and/or cognitive techniques as these are included in both sets of guidelines as strongly recommended.

Trauma-focused CBT is based on cognitive and behavioral models that tend to draw from other CBT theories, such as PE and CPT. For example, Ehlers and Clark (2000) proposed that individuals with PTSD hold excessively negative appraisals of the trauma and that their autobiographical memory of the trauma is characterized by poor contextualization, strong associative memory and strong perceptual priming, which leads to involuntary reexperiencing of the trauma. Ehlers and Clark suggest that individuals with PTSD engage in problematic behavioral and cognitive strategies that prevent them from changing negative appraisals and trauma memories. Thus, goals of this treatment include modifying negative appraisals, correcting the autobiographical memory, and removing the problematic behavioral and cognitive strategies. Kubany et al. (2004) suggest that guilt-associated appraisals may evoke negative affect and may be paired with images or thoughts of the trauma. These guilt appraisals may repeatedly recondition memories of the trauma with distress and may lead to tendencies to suppress or avoid trauma-related stimuli.

Trauma-focused CBT typically includes both behavioral techniques, such as exposure, and cognitive techniques, such as cognitive restructuring. CBT that includes exposure to the traumatic memory uses imaginal exposure, writing the traumatic narrative, or reading the traumatic memory out loud (Marks et al., 1998; Kubany et al., 2004; Ehlers et al., 2005). CBT that includes exposure to trauma-related stimuli typically uses *in vivo* exposure (Kubany et al., 2004) or teaching patients to identify triggers of re-experiencing and practice discrimination of “then vs. now” (Ehlers et al., 2005). Cognitive restructuring focuses on teaching patients to identify dysfunctional thoughts and thinking errors, elicit rational alternative thoughts, and reappraise beliefs about themselves, the trauma, and the world (Marks et al., 1998; Kubany et al., 2004; Ehlers et al., 2005). A CT targeting PTSD among battered women focused specifically on CT for trauma-related guilt in three phases: guilt issue assessment, guilt incident debriefings and CT (Kubany et al., 2004).

Consistent with the recommendations of the guidelines, research supports the effectiveness of trauma-focused CBT for PTSD. CBT has been shown to be more effective than a waitlist (Power et al., 2002), supportive therapy (Blanchard et al., 2003) and a self-help booklet (Ehlers et al., 2003). Researchers have compared different components of CBT (i.e., imaginal exposure, *in vivo* exposure, cognitive restructuring) with some mixed results. Marks et al. (1998) compared exposure therapy (that included five sessions of imaginal exposure and five sessions of *in vivo* exposure), cognitive restructuring, combined exposure therapy and cognitive restructuring, and relaxation in an RCT. Exposure and cognitive restructuring were each effective in reducing PTSD symptoms and were superior to relaxation. Exposure and cognitive restructuring were not mutually enhancing when combined. Bryant et al. (2008) compared imaginal exposure alone, *in vivo* exposure alone, imaginal and *in vivo* exposure, and imaginal, *in vivo*, and cognitive restructuring. In contrast to Marks et al. (1998), Bryant et al. (2008) found the treatment condition with both exposure components and cognitive restructuring had the largest effect size and resulted in fewer patients with PTSD at a 6-month follow-up. Regarding loss of diagnosis, 61% to 82.4% of participants treated with CBT lost their PTSD diagnosis and 26% more CBT participants than waitlist or supportive counseling achieved loss of PTSD diagnosis (Jonas et al., 2013).

### Dropout, Side Effect and Adverse Effects

One common concern with trauma-focused treatment is dropout and rates of dropout appear to be similar across PE, CPT and trauma-focused CBT (Hembree et al., 2003). A substantial minority of individuals drop out of PTSD treatment (e.g., Imel et al., 2013). Imel et al. (2013) conducted a meta-analysis of treatment dropout in PTSD treatment. The aggregate proportion of dropout across all active treatments was 18.28%, however, there was a large amount of variability across studies. The dropout rate varied between active interventions for PTSD across studies, but the differences were primarily driven by differences between studies. In addition, an increase in trauma focus did not predict an increase in the dropout rate. Imel et al. (2013) did find evidence across three relatively large trials that dropout is lower in present centered therapy (PCT; 22%) compared to trauma specific treatments (36%).

Unfortunately, few studies explicitly report on side effects and adverse effects of PTSD psychotherapy (Cusack et al., 2016). The American Psychological Association (2017) guidelines recommends that research be conducted on side effects. When examining the results of large controlled trials there is no evidence that trauma-focused treatments are associated with a relative increase in adverse side effects (American Psychological Association, 2017; VA/DoD Clinical Practice Guideline Working Group, 2017). Clearly more research should examine and report on side effects and adverse effects of PTSD treatment.

## Implications and Future Directions

PE, CPT and trauma-focused CBT have been strongly recommended as treatments for PTSD in treatment guidelines by the APA and the VA/DoD. Each of these treatments have a large evidence base supporting their effectiveness in treating PTSD. Although exposure-based therapies have the largest and strongest research evidence base (Cusack et al., 2016), research and meta-analyses comparing PE, CPT and trauma-focused CBT do not find that one treatment outperforms the other (Resick et al., 2002, 2008; Powers et al., 2010; Cusack et al., 2016).

The guidelines and strong research evidence suggest that PE, CPT and trauma-focused CBT should be the first line of treatment for PTSD whenever possible, considering patient preferences and values and clinician expertise. Research examining patient preferences suggests that individuals prefer PE, CPT and trauma-focused CBT to other treatments. Analog studies have demonstrated that participants have preferences for CT and exposure therapy over psychodynamic psychotherapy, EMDR, and therapies using novel technologies (e.g., virtual reality, computer-based therapy; Tarrier et al., 2006; Becker et al., 2007). In addition, results from studies examining clinical samples show that patient prefer psychotherapy, such as PE and CBT, to medication (Angelo et al., 2008; Feeny et al., 2009; Zoellner et al., 2009). Findings are similar among veteran and military samples, with soldiers showing greater preference for PE and virtual reality exposure (VRE) to paroxetine or sertraline (Reger et al., 2013) and veterans in a PTSD specialty clinic showing greater preference for CPT to other psychotherapies, PE to nightmare resolution therapy and PCT, and both PE and cognitive-behavioral conjoint therapy were preferred to VRE (Schumm et al., 2015).

The recommendations to use these treatments by the guidelines has not been without controversy in the provider community, as evidenced by online petitions against the APA guidelines (there is also a petition supporting the guidelines). Those who petition these guidelines may be concerned that trauma-focused treatments could pose a risk to some patients because of distress elicited by focusing on the trauma memory, may limit providers’ ability to get reimbursed for other types of treatment, or they may believe that RCTs lead to false conclusions (for a rebuttal, see McKay, 2017; Shedler, 2017). However, as stated above, there is no evidence that trauma-focused treatments are associated with a relative increase in adverse side effects (American Psychological Association, 2017; VA/DoD Clinical Practice Guideline Working Group, 2017). In addition, although RCTs cannot answer all questions in clinical psychology science, they do eliminate more sources of error (e.g., placebo effect, confirmation bias) than other research designs, such as naturalistic or observational studies. Thus, dissemination of information about effective treatments, benefits and harms related to treatment, and effective research methodology to treatment providers who work with individuals with PTSD is imperative. There is also concern that these trauma-focused treatments may not be as effective among military samples (Steenkamp et al., 2015; Steenkamp, 2016). According to a review of trauma-focused treatment among military samples, approximately 60% to 72% of military patients retained PTSD diagnosis after treatment (Steenkamp et al., 2015). However, this rate was lower than comparison groups including waitlist and PCT (range 74%–97%), within-group posttreatment effect sizes for CPT and PE were large, and 49%–70% of patients receiving CPT or PE attained clinically meaningful symptom improvement (defined as a 10–12 point decrease in interviewer or self-report symptoms (Steenkamp et al., 2015). Findings from this review support the recommendation of the guidelines that PE, CPT and trauma-focused CBT should be the first line of treatment for PTSD and also suggest that outcomes from these treatments can be improved.

Future directions in PTSD treatment research include identifying ways to enhance effective treatments including among particular populations (e.g., military), further examination of treatments that are “recommended” rather than “strongly recommended”, keeping individuals engaged in treatment (i.e., reducing dropout), and determining individual factors predicting response/nonresponse. Avoidance symptoms are a core feature of  PTSD and maintain PTSD over time. Thus, it is not surprising that the dropout rate for PTSD treatment is high across treatment modalities. In addition, a portion of individuals do not respond adequately to PTSD treatment. One potential future direction is medication-enhanced psychotherapy for PTSD. Medication could potentially strengthen learning and memory, inhibit fear, and facilitate therapeutic engagement (Dunlop et al., 2012). Research is beginning to examine pharmacological agents to enhance response to trauma-focused therapies such as MDMA, D-cycloserine and the neuropeptide oxytocin (e.g., Mithoefer et al., 2011; de Kleine et al., 2012; Koch et al., 2014; Rothbaum et al., 2014). Non-pharmacological enhancement of therapy is also being explored such as rTMS (Kozel et al., 2018), exercise (Rosenbaum et al., 2015), and other cognitive training (Fonzo et al., 2017). Another potential avenue to increase engagement and reduce dropout is through use of intensive treatment programs, in which patients attend massed multiple sessions within a short period of time (e.g., one or 2 weeks) instead of weekly sessions spaced over several months. These types of programs are beginning to be evaluated with promising results (e.g., Harvey et al., 2017; Foa et al., 2018; Hendriks et al., 2018) and report excellent retention rates (90%–100%).

Further research on particular PTSD treatments is needed. As research continues to transition to the utilization of DSM-5 criteria, it will be essential to update the guidelines informed by the new criteria as this new conceptualization could impact the measurement and efficacy of these treatments. Examining biomarkers of PTSD, treatment response, and precision medicine, i.e., matching treatment to the individual, are the wave of the future. We need to compare interventions and determine if any treatment approaches are more or less effective for particular groups of people. Finally, further research is needed to develop new treatment approaches that are effective and acceptable to PTSD sufferers, as recommended in the 2014 IOM report (Institute of Medicine, 2014).

## Conclusion

The guidelines put forth by the VA/DoD and the APA in 2017 are recommendations for providers who treat individuals with PTSD and both strongly recommend PE, CPT and trauma-focused CBT. Each of these treatments has a large evidence base showing their effectiveness. These treatments are all trauma-focused, which means they directly address memories of the traumatic event or thoughts and feelings related to the traumatic event. Treatments with the strongest evidence should be the first line of treatment for PTSD whenever possible, with consideration of patient preferences and values and clinician expertise.

## Author Contributions

LW, KS and BR discussed and conceived the topic and content of the review. LW and KS drafted the manuscript. BR wrote portions that appear throughout the manuscript. All authors provided critical revisions and approved the final manuscript.

## Conflict of Interest Statement

The authors declare that the research was conducted in the absence of any commercial or financial relationships that could be construed as a potential conflict of interest. The reviewer ED and handling editor declared their shared affiliation at the time of review.
